# Social situations differ in their contribution to population‐level social structure in griffon vultures

**DOI:** 10.1002/ece3.10139

**Published:** 2023-06-02

**Authors:** Nitika Sharma, Nili Anglister, Orr Spiegel, Noa Pinter‐Wollman

**Affiliations:** ^1^ Department of Ecology and Evolutionary Biology University of California Los Angeles Los Angeles California USA; ^2^ School of Zoology, Faculty of Life Sciences Tel Aviv University Tel Aviv Israel

**Keywords:** collective movement, communal roosting, GPS‐telemetry, *Gyps fulvus*, social environment, social foraging, social network analysis

## Abstract

Social relationships among animals emerge from interactions in multiple ecological and social situations. However, we seldom ask how each situation contributes to the global structure of a population, and whether different situations contribute different information about social relationships and the position of individuals within the social fabric. Griffon vultures (*Gyps fulvus*) interact socially in multiple situations, including communal roosting, joint flights, and co‐feeding. These social interactions can influence population‐level outcomes, such as disease transmission and information sharing that determine survival and response to changes. We examined the unique contribution of each social and ecological situation to the social structure of the population and individuals' positions within the overall social network using high‐resolution GPS tracking. We found that the number of individuals each vulture interacted with (degree) was best predicted by diurnal interactions—both during flights and on the ground (such as when feeding). However, the strength of social bonds, that is, the number of interactions an individual had (strength), was best predicted by interactions on the ground—both during the day (e.g., while feeding) and at night (e.g., while roosting) but not by interactions while flying. Thus, social situations differ in their impact on the relationships that individuals form. By incorporating the ecological situations in which social interactions occur we gain a more complete view of how social relationships are formed and which situations are important for different types of interactions.

## Graphical abstract

Social relationships among animals emerge from interactions in multiple ecological and social situations. However, we seldom ask how each situation contributes to the global structure of a population, and whether different situations contribute different information about social relationships and the position of individuals within the social fabric. By incorporating the ecological situations in which social interactions of griffon vultures occur we gain a more complete view of how social relationships are formed and which situations are important for different types of interactions.

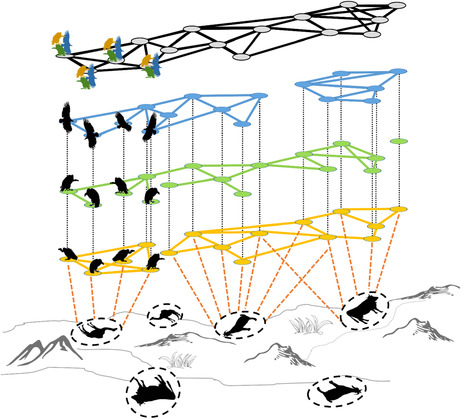

## 1 INTRODUCTION

The social relationships among animals emerge from interactions in multiple ecological and social situations. Relationships can result from both affiliative interactions, such as grooming and food sharing, and agonistic encounters, such as direct aggression and indirect supplanting (Whitehead, 2008). Social structures have important population‐level outcomes such as disease dynamics and the spread of information. Traditionally, different types of interactions have been studied either separately or in aggregate, without distinguishing among them (Croft et al., 2008; Krause et al., 2010; Pinter‐Wollman et al., 2014; Wey et al., 2008). However, observed social relationships are a product of interactions that take place in different social and ecological situations (Dragić et al., 2021; Finn et al., 2019; Fischer et al., 2017). Social animals can benefit from certain types of associations by gaining knowledge about the location, availability, and quality of resources (Dall et al., 2005; Giraldeau & Caraco, 2018). However, the potential costs of sociality, such as fast depletion of resources, competition over mates, and increased exposure to pathogens all impact social dynamics (Evans et al., 2020; Silk, 2007). The balance between the costs and benefits of sociality can determine how each social situation (sometimes referred to as “context”) contributes to the global social structure. Thus, a closer examination of interactions that occur in different social situations and their relative contribution to the social structure of a population may provide more accurate information about the mechanisms that underlie social structures and the function of sociality in population‐level processes (Silk et al., 2018).

Each social and ecological situation may contribute differently to the position of an individual in a society because individuals may differ in how much they engage with others in each social situation. For example, certain individuals may be important for stabilizing a social group (Flack et al., 2006), or are important in foraging situations, leading groups to scarce resources (Brent et al., 2015; Foley et al., 2008; Mccomb et al., 2001). However, those individuals may play a more peripheral social role in other situations, such as caring for offspring, or group defense. The common approach of aggregating all interaction types makes it impossible to distinguish between an individual that has many interactions in one particular situation and an individual that has few interactions with unique individuals in a diverse set of situations. Thus, treating social interactions in different situations as components of a unified social structure can produce unexpected inferences about the role of individuals in their society (Finn et al., 2019). For example, in a recent study of paper wasps, the potential of an individual to become a queen was revealed only when social interactions in four different situations were considered simultaneously. However, it was not revealed when all interactions (that were included in the multilayer network) were aggregated without distinguishing among situations, or when interactions in each situation were considered separately (Sharma et al., 2022). Similarly, in primate societies, certain individuals were identified as important in the social structure only when multiple social situations were considered together, but not when each social situation was examined separately (Smith‐Aguilar et al., 2019) and the effect of removing an important individual cascade across social situations (Barrett et al., 2012). By considering different social situations, certain situations emerge as more important in shaping the sub‐structure of the society than others (Smith‐Aguilar et al., 2019). Thus, uncovering which social and ecological situations influence individuals' social roles in each situation has important implications for determining survival and exposure to pathogens (Vanderwaal et al., 2016) as well as social foraging (Boogert et al., 2014), which are important for wildlife conservation and management.

**FIGURE 1:**
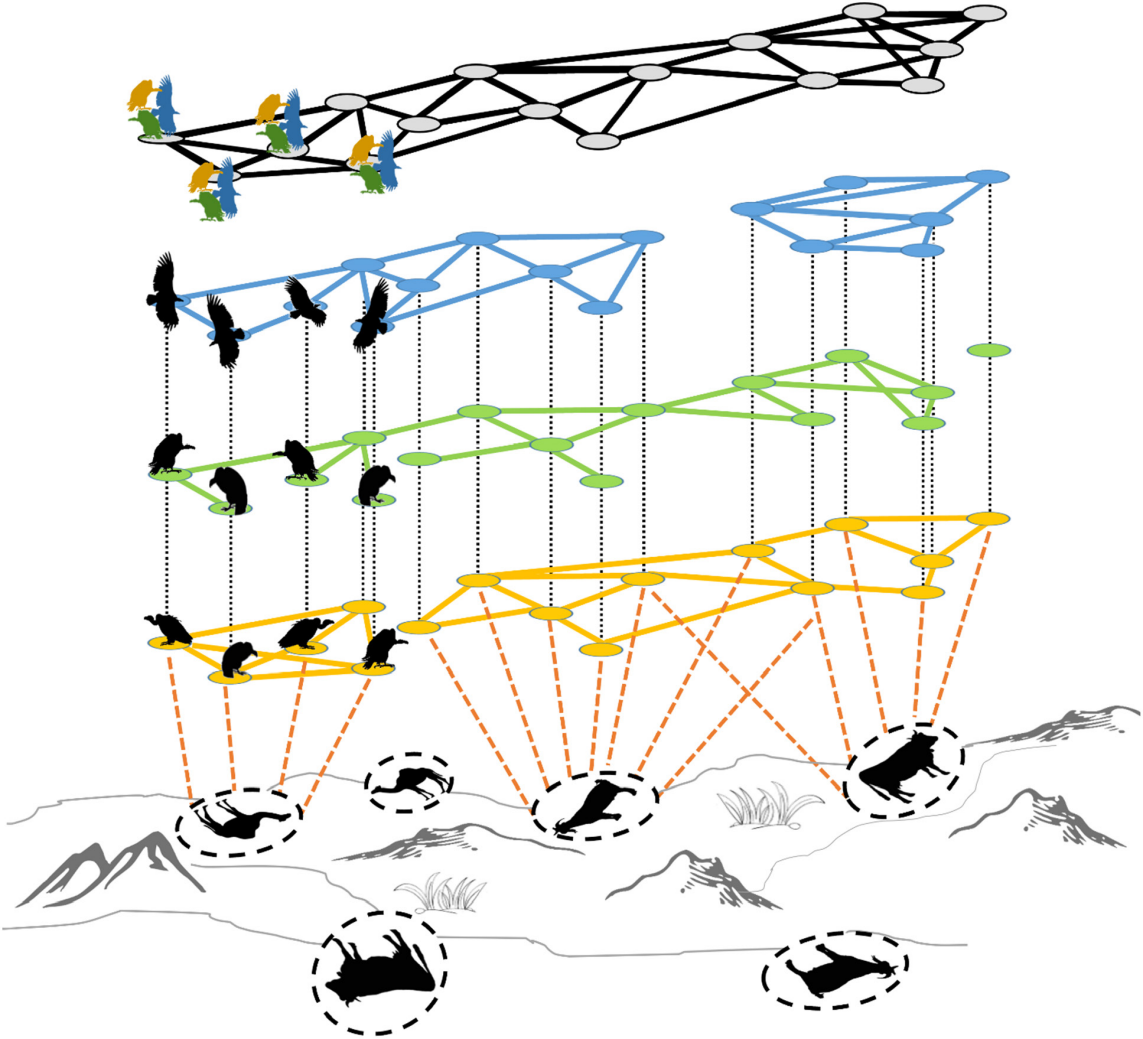
Social networks across multiple situations. A hypothetical example of social interactions among vultures in different social situations: co‐flying in blue, nocturnal ground interactions (i.e., co‐roosting) in green, and diurnal ground interactions (e.g., co‐feeding) in yellow. Solid lines within each social situation indicate interactions within the social situation and black‐dotted lines between social situations connect occurrences of the same individual. Dashed orange lines connect individuals to food sites to show how spatial proximity can be used to infer social interactions, for example when co‐feeding. An aggregate network at the top, in gray, combines all interactions from the different social situations.

Griffon vultures (*Gyps fulvus*) interact in different social situations to share social information about the location of roosts and feeding sites (Figure 1). Like most other vulture species, griffons are large obligate scavengers that search for and consume large carcasses (Houston, 1974). Because carcasses are an unpredictable resource, griffons rely heavily on social information and recruitment to locate food (Cortés‐Avizanda et al., 2014; Deygout et al., 2010; Jackson et al., 2008; Spiegel, Getz, & Nathan, 2013). Recruitment to food results in local enhancements and feeding aggregations of tens of individuals, which may share food and/or engage in aggressive interactions (Carrete et al., 2010; Mundy, 1992). Griffons use communal roosts and nest in colonies, which can serve as information centers for locating resources (Buckley, 1996; Harel et al., 2017). Thus, interactions in different situations may provide different information and contribute in different ways to the relationships that vultures form. Furthermore, individuals may differ in their need for food as well as in their knowledge about the location and quality of current resources, which depend on their recent movements and their interactions with conspecifics in different situations (Spiegel, Harel, et al., 2013). For example, if information about food location is obtained through co‐flying (Cortés‐Avizanda et al., 2014), vultures that spend much time flying with others might have greater access to food than those who tend to fly in smaller groups or alone. In contrast, if information sharing at the roost is more important for locating food, then individuals that roost with more individuals and/or with better‐informed ones will have greater access to food (Harel et al., 2017). In addition, interactions on the ground (e.g., when roosting or feeding) may expose vultures to information about social status and potential mates, but also to pathogens. Thus, individuals that spend more time in ground‐based interactions might have more exposure to certain information and disease compared to those that interact predominantly during flight.

The goal of our study is to examine how different behavioral situations contribute to the social structure of a vulture population and to determine how individuals differ in their social position based on the social situations (contexts) in which they interact. Specifically, we consider three social situations (contexts): diurnal interactions on the ground (e.g., while co‐feeding), diurnal interactions in the air (co‐flying), and nocturnal interactions on the ground (e.g., while co‐roosting). We ask if individuals that have a central role in one social situation carry over their social role to other situations. We further ask if social situations contribute in different ways to the population‐level social structure. We hypothesize that the social and ecological context of an interaction will impact the way in which it structures animal relationships. Specifically, we predict that social situations with brief interactions, such as co‐flying, will have a lower impact on the strength of social bonds compared to situations in which interactions are long, such as those that occur on the ground because vultures spend more time on the ground than in the air during the day (Fluhr et al., 2021). We further predict that social situations in which movements are shorter (i.e., on the ground) will result in fewer unique interactions relative to situations in which vultures move larger distances (i.e., when flying). Disentangling the role of social interactions in different situations, both at the individual and the population levels, will shed light on the complexities of animal societies and the ecological implications of social interactions.

## 2 METHODS

### 2.1 Animal capture and tagging

**FIGURE 2:**
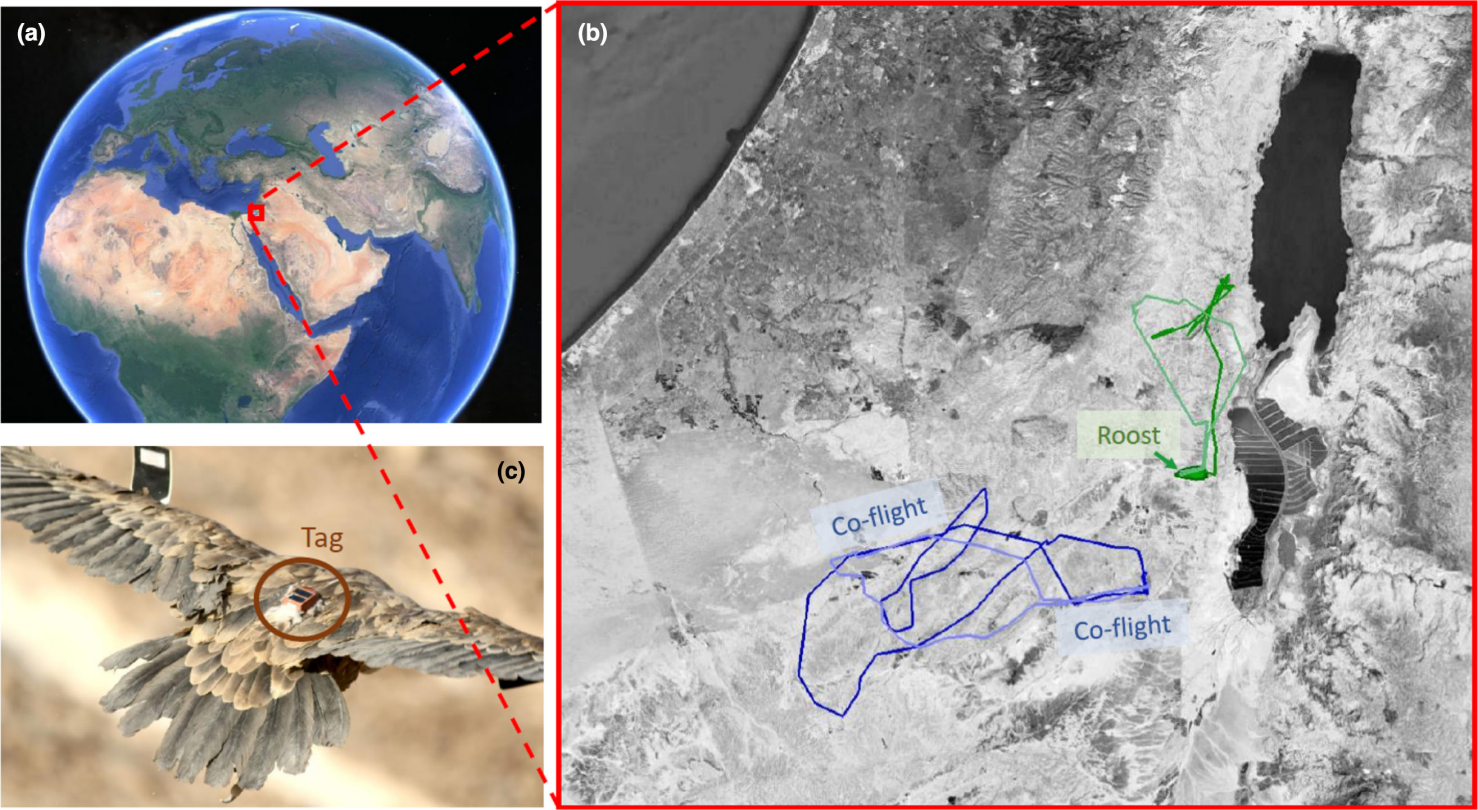
Inferring interactions from movements. (a) Location of study site – small red square – on a Satellite image of Earth (from GoogleEarth). (b) Satellite image (from GoogleEarth) of the study area in southern Israel (the Judean and Negev deserts) with example trajectories of vultures that interacted in two different social situations. Blue lines are movement trajectories of vultures that engaged in co‐flight. The word “co‐flight” appears next to the two regions of their trajectories that overlapped in time and space—deeming them co‐flight interactions. Green lines are movement trajectories of two vultures that roosted at the same roost overnight and therefore are considered to have a nocturnal ground interaction. The small green polygon to the bottom right of the word “Roost” is the roost in which they both spent the night. Note that these two vultures did not engage in co‐flight interactions. (c) Photo of a griffon vulture with a GPS tag attached to its back (circled) and wing tags (photo credit: Tovale Solomon).

Because the Israeli population of griffon vultures is considered regionally critically endangered (Mayrose et al., 2017), the Israeli Nature Protection Authority (INPA) operates a large‐scale management program that we collaborated with. This program includes routine captures of free‐ranging individuals with walk‐in traps and the release of captive‐bred or imported griffons. Captured birds (~100 annually, including frequent re‐traps) are banded and marked with patagial tags for field identification, and a subset is equipped with tracking devices. During September–November 2020 we tagged 47 griffons with Ornitela OrniTrack GPS‐GSM tags (50 g, Figure 2c), in a leg‐loop harness configuration (Anderson et al., 2020). These tags record the location, speed, and altitude of individuals every 10 minutes only during the day to preserve battery power, because vultures are diurnal. The high temporal resolution and spatial accuracy (errors of a few meters compared to vast movements across tens of kilometers daily) allow us to determine the social interactions of vultures in different situations based on spatial proximity, as detailed below.

We restricted our examination of vulture social interactions to the breeding season (from December 2020 to June 2021) because that is when vultures remain local in Israel and the close surroundings (mostly within southern Israel and adjacent Jordan). We avoided the non‐breeding season (summer and fall) because that is when vultures tend to go on long‐range forays, spreading beyond their local home range throughout the entire Mediterranean (e.g., from Sudan through Saudi Arabia to Turkey; Spiegel et al., 2015). During these forays, they may often interact with untagged individuals, preventing reliable representation of their social interactions. In contrast, during the breeding season, they remain locally and we are likely to capture most social interactions from the movement data recorded by the tags because a very high proportion of the population is tagged. In our analysis, we only used data from individuals whose tags provided locations for more than a third of the breeding season (i.e., for >71 days) and who remained within the local geographic region of the study population in southern Israel (i.e., within 400 km of where they were tagged) throughout the breeding season. We used these temporal and geographic restrictions to increase the likelihood of capturing the majority of social interactions in the population. After applying these temporal and geographic filtering, we remained with rich movement data for 29 vultures, which are approximately 15% of the Israeli vulture population and 20% of the population in southern Israel. Tracking 20% of the effective population provides approximately 75% accuracy for estimating the network measures we examined, according to simulation studies (Silk et al., 2015).

### 2.2 Constructing social networks from spatial data

To examine social interactions in different situations we focused on three social situations: co‐flying, diurnal interactions on the ground (e.g., while co‐feeding), and nocturnal interactions on the ground (namely while co‐roosting). All social interactions were inferred from movement data obtained from the GPS tags, based on movement speed and spatial proximity (see number of interactions over time in Figure S1). To examine the social structure that resulted from these interactions, for each social situation, we created an undirected weighted interaction network (Croft et al., 2008; Wey et al., 2008). In each network, nodes denote individually tagged vultures and edges connect vultures that interacted in a particular social situation, based on the details below. To scale the number of interactions within each situation, edge weights represented the association strength between each pair of vultures measured as a simple ratio index (SRI). An SRI divides the number of times two vultures were observed together (as defined below) by the total number of times they could have interacted—that is, times in which they both had a GPS location recorded (Ginsberg & Young, 1992).

#### 2.2.1 Co‐flying

Vultures were considered flying if they were both moving faster than 5 m/s (Spiegel, Harel, et al., 2013). Owing to their vision, vultures can see each other from afar, and potentially recognize each other (Pennycuick, 1971; Spiegel, Getz, & Nathan, 2013). We, therefore, deemed individuals flying within 1000 m of each other for at least two consecutive GPS fixes (which are separated by 10 min) to have a co‐flight interaction (Figure 2).

#### 2.2.2 Nocturnal ground interactions

Vultures nest and roost on high cliffs and tend to aggregate at communal roosts. Individuals were considered to interact on the ground during the night if they co‐occurred at the same roost overnight. Roosts were spatially defined as polygons on a map‐ (see example roost polygon in Figure 2 and Harel et al. (2017)) shaped according to geographic features, such as dry streams or cliffs, where vultures are known to roost. The area of the roost polygons was 783.5 ± 1751.3 m^2^ (mean ± SD), which falls well within the perceptual range of vultures. To account for poor reception within the canyons serving as roosts, and for the vultures’ diurnal activity, we associated vultures with their nightly roost polygons using either their last position of the day or their first position on the following morning; if neither of these two locations fell within a roost polygon, we used the average Euclidean distance between these two positions to assign a vulture to a roost. Vultures occasionally roost outside of communal roosts, so if this average position did not fall within a roost, we did not assign those locations to any roost.

#### 2.2.3 Diurnal ground interactions

Vultures were considered interacting on the ground during the day if they were not flying (i.e., ground speed slower than 5 m/s; Spiegel, Harel, et al., 2013), and their locations were within 50 m of each other for at least two consecutive GPS fixes (which are separated by 10 min). We excluded any interactions inside known roosts during the day. Therefore, these diurnal ground interactions likely represent interactions while feeding and joint sunbathing at feeding sites.

#### 2.2.4 Aggregate networks

To combine interactions from all social situations in a single aggregate network, we summed the edge weights from all three social situations (co‐flight, diurnal, and nocturnal ground interactions) for each pair of individuals and then recalculated the measures below.

### 2.3 Social network analysis

#### 2.3.1 Quantifying role of individuals

To determine the role of individuals in the social structure, we calculated standard biologically relevant centrality measures (Krause et al., 2010; Pinter‐Wollman et al., 2014; Wey et al., 2008) for each individual in each social situation and in the aggregate network:

##### Degree

To determine how many other vultures each individual might impact, we quantify degree—the number of unique individuals that a vulture interacted with.

##### Strength

To determine how impactful interactions might be we measure interaction strength—the intensity of interactions of an individual, calculated as the sum of the weights of all the edges that reach a node. Higher values indicate more strongly connected individuals.

##### Page rank

To determine how far the social influence of an individual might reach we measure PageRank—a score given to a node based on second‐order interactions, such that nodes connected to well‐connected individuals have a higher score. For more information on the algorithm, see the documentation of the page_rank() function in the igraph package (Csardi & Nepusz, 2006).

#### 2.3.2 Quantifying contributions of social situations to population structure

To quantify the relative contribution of each social situation to the position of individuals in the population's social structure (the aggregate network), we calculated the Spearman's correlation (*ρ*) for each centrality measure between each social situation and the aggregate network. For example, we correlated the degree of individuals in the co‐flight network with the degree of individuals in the aggregate network, the strength in the co‐flight network with strength in the aggregate, etc. These comparisons resulted in nine correlations (3 indices × 3 social situations). To further determine the contribution of each social situation to the population's social structure we asked whether the observed correlation coefficients differed from those expected by chance by comparing observed ρ values to those extracted from reference models. We created 10,000 reference (randomized) networks using node permutations (Hobson et al., 2021). In each iteration, the node IDs within each of the three social situations were permuted without replacement and the three centrality measures were calculated for each situation. By permuting only node IDs, we maintained the observed network structure while breaking the relationship between social positions within and across situations. For each iteration, the reference aggregate network was created by combining the permuted networks of the three social situations. The three centrality measures were then calculated for the aggregate network. We then computed the Spearman's correlation for each centrality measure between the social situations and the aggregate network for each of the permutation iterations. We determined statistical significance by computing a *p*‐value as the proportion of iterations in which the observed Spearman's correlation coefficient (*ρ*) was larger or smaller than 95% of the *ρ* coefficients in the permutated data.

Analysis was conducted in R version 3.4 (R Core Team, 2013). Network analysis was conducted using the “igraph” R package (Csardi & Nepusz, 2006) and Muxviz (De Domenico et al., 2015). Data are provided as part of the supplementary material and the analysis code is available on GitHub (https://github.com/NitikaIISc/VulturesMovementAnalysis_manuscript1).

## 3 RESULTS

**FIGURE 3:**
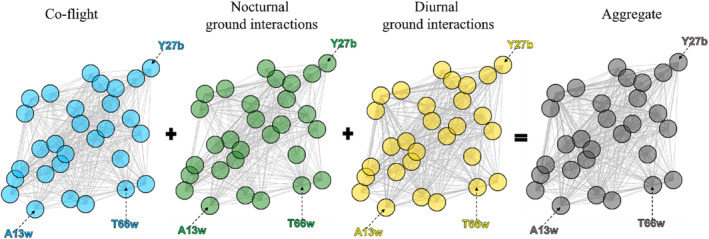
Social networks in different social situations. Networks of 29 individually tagged vultures during the breeding season (December 2020–June 2021). From left to right the situations include co‐flight (blue), nocturnal ground interactions (green), diurnal ground interactions (yellow), and an aggregate network that combines interactions in all three situations (gray). Nodes depict individual vultures and the position of each node (individual) is maintained in all four networks. As an example, the identities of three individuals are specified in all four networks. Lines connecting nodes indicate that individuals interacted within a social situation, and line thickness corresponds to association strength.

**FIGURE 4:**
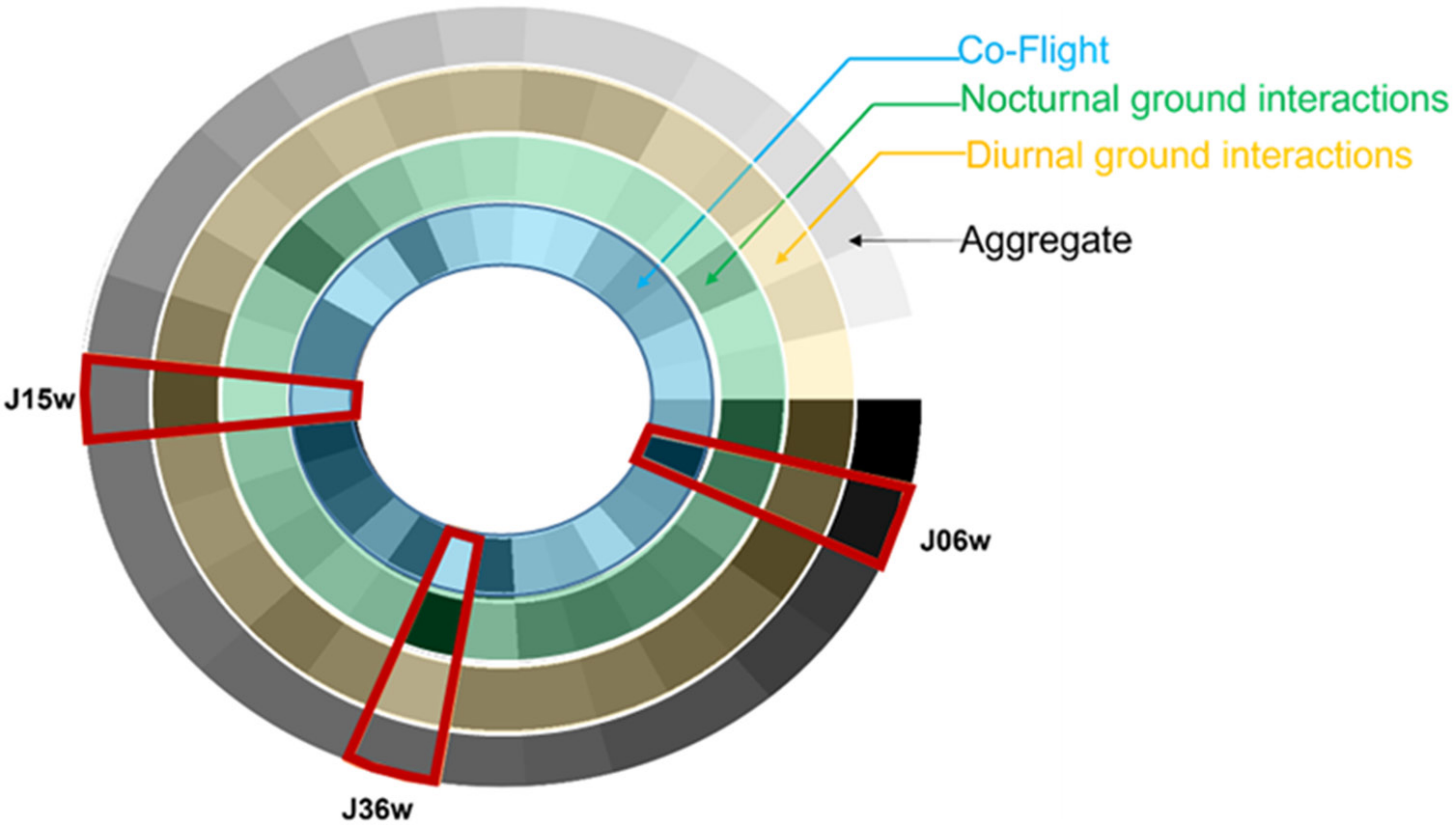
Individuals differ in their social position across social situations. Annular representation of the interaction strength of 29 tagged vultures in the breeding season of 2021. Three rings represent the three social situations: co‐flight (blue hue), nocturnal ground interactions (green hue), and diurnal ground interactions (yellow hue). The outer ring represents the aggregate network (gray hue). Darker shades indicate a higher rank of interaction strength. Each slice in the ring corresponds to one individual. Some individuals may be important in one social situation but not in others. For example, of the three individuals that are highlighted, J15w has a strength that is highly ranked in the diurnal ground interactions but not in the other social situations. Similarly, J36w is highly ranked in the nocturnal ground interactions but not in other social situations. Finally, individual J06w is highly ranked in co‐flight interactions but not in other situations. Similar plots for degree and PageRank are provided in Figure S2.

All 29 individuals interacted with at least one other vulture in all three social situations: co‐flight, diurnal, and nocturnal ground interactions (Figure 3, Table S1). The centrality of individuals differed across social situations. Individuals differed in their importance across social situations in terms of their degree, strength, and PageRank. For example, a vulture that interacted with multiple vultures while co‐flying did not necessarily interact with as many individuals while on the ground (Figure 4).

**FIGURE 5:**
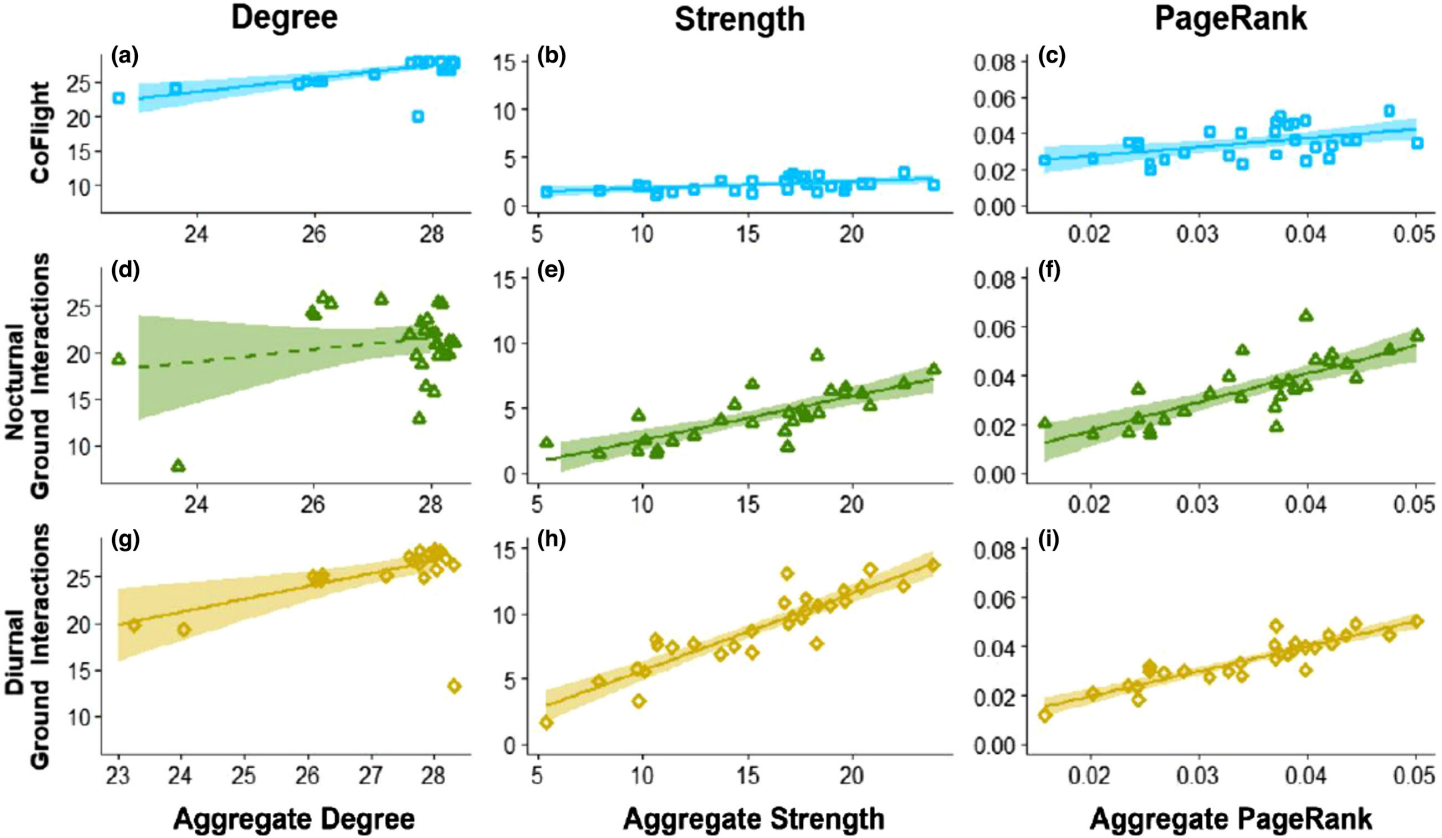
Relationship between centrality in the aggregate network and each social situation. Correlation between centrality measures (degree [a, d, g], strength [b, e, h], and PageRank [c, f, i]) in the aggregate network (*x* axes) and each of the three social situations (*y* axes): (a–c) co‐flight, (d–f) nocturnal ground interactions, and (g–i) diurnal ground interactions. Each point is an individual vulture and points were jittered (0.4) along the *x* and *y* axes to improve readability in the plots of degree (a, d, g). Lines are linear regressions and the shading around the line is the 95% confidence interval computed using ggscatter() function of the ggpubr R package (Kassambara, 2023) with the lower confidence limit being 1 ± 0.95/2 percentiles.

The centrality of individuals in the aggregate network did not necessarily reflect their centrality in each social situation. The degree of individuals in co‐flight and diurnal ground interactions were positively correlated with the degree in the aggregate network (Spearman's correlation: co‐flight: *ρ =* 0.784, *p*‐value < .0001, and diurnal ground interactions: *ρ =* 0.681, *p*‐value < .0001; Figure 5a,g, Table 1). However, the degree in nocturnal ground interactions was not significantly correlated with the degree in the aggregate network (Spearman's correlation: *ρ =* −0.17, *p*‐value = .377; Figure 5d, Table 1). Both strength and PageRank in each of the three social situations were positively correlated with the strength and PageRank in the aggregate network (Spearman correlations for *strength* of aggregate with: co‐flight *ρ =* 0.437, *p*‐value = .019; nocturnal ground interactions *ρ =* 0.799, *p*‐value < .0001; and diurnal ground interactions *ρ =* 0.888, *p*‐value < .0001; Figures 5b,e,h and Spearman correlations for *PageRank* of aggregate with: co‐flight *ρ =* 0.391, *p*‐value = .037; nocturnal ground interactions *ρ =* 0.8, *p*‐value < .0001; and diurnal ground interactions *ρ =* 0.89, *p*‐value < .0001, Figure 5c,f,i, Table 1).

**TABLE 1:** Statistical output of Spearman's correlation between each social situation and the aggregate network for the three network measures we tested.

	Degree	Strength	PageRank
Spearman's *ρ*			
Co‐flight	0.784	0.437	0.391
Nocturnal ground interactions	−0.17	0.799	0.8
Diurnal ground interactions	0.681	0.888	0.89
*p*‐Value			
Co‐flight	**<.0001**	**.019**	**.037**
Nocturnal ground interactions	.377	**<.0001**	**<.0001**
Diurnal ground interactions	**<.0001**	**<.0001**	**<.0001**

*Note*: Values in bold indicate statsitical significance.

**FIGURE 6:**
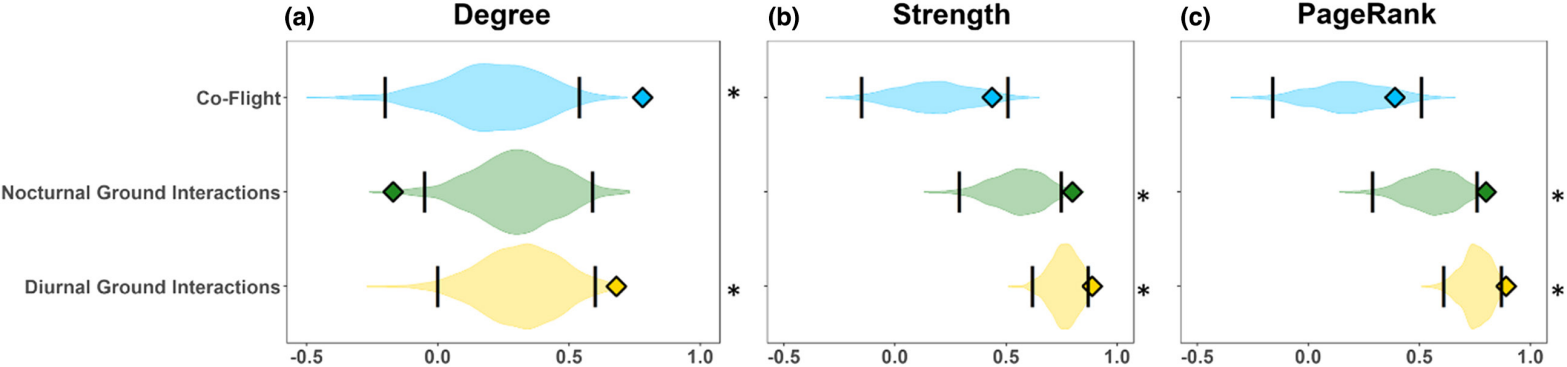
Comparing observed and expected correlations. Observed (diamonds) and randomized reference distribution (violins) of Spearman's correlation coefficient (*ρ*) between three centrality measures: degree (a), strength (b), and PageRank (c) in each of the three social situations: co‐flight (blue), nocturnal ground interactions (green), and diurnal ground interactions (yellow). Vertical black lines on the violin plots depict the 95% quantiles of the reference distribution. Asterisks to the right of each plot denote statistically significant differences between the observed values and chance distribution using a two‐sided test. Violin plots were created using ggplot2 (Wickham, 2016).

The correlation between centrality in the aggregate network and each social situation was often different than expected by chance when compared with the reference models. Degree both in the co‐flight and diurnal ground interactions was positively correlated with the degree in the aggregate more than expected by chance (Permutation test: co‐flight *p*‐value < .0001 and diurnal ground interactions *p*‐value = .012; Figure 6a). The degree in nocturnal interactions was negativly correlated with the degree in the aggregate network more than expected by chance (Permutation test: *p*‐value =  .0182; Figure 6a). Strength in the nocturnal and diurnal ground interactions was positively correlated with strength in the aggregate network more than expected by chance (nocturnal ground interactions: *p*‐value = .0276 and diurnal ground interactions: *p*‐value = .0076; Figure 6b). However, although the relationship between strength in co‐flight and strength in the aggregate network were correlated (Figure 5b) this correlation did not differ significantly from chance expectation (co‐flight: *p*‐value = .175; Figure 6b). PageRank in the nocturnal and diurnal ground interactions was positively correlated with PageRank in the aggregate network, more than expected by chance (nocturnal ground interactions: *p*‐value = .0274 and diurnal ground interactions: *p*‐value = .0028, Figure 6c). However, the positive relationship between PageRank in co‐flight and PageRank in the aggregate network (Figure 5c) was not significantly different than expected by chance (co‐flight: *p*‐value = .2688; Figure 6c).

## 4 DISCUSSION

We found that the way in which social situations differ in their contribution to the social structure of griffon vultures relates to their ecological context. There was no single individual that ranked the highest in all centrality measures or across all social situations (Figure 4, Figure S2). Furthermore, the centrality of individuals in the aggregate network did not always reflect the centrality of individuals in each of the social situations (Figure 5). Finally, social situations differed in their contribution to the different centrality measures, with some being more important for the number of individuals a vulture interacted with and others being more important for the strength of the social interaction (Figure 6), as we predicted.

Individuals differed in their social importance across social situations. While some individuals had a similar centrality rank in all three social situations, others did not. Thus, the social role of an individual does not necessarily carry over across situations. The differential social importance of individuals across social situations may have implications for population‐level processes. For example, individuals that interact with many others (high degree) while co‐feeding might spread pathogens broadly during feeding interactions, however, if they do not interact with many individuals while roosting, their impact on pathogen spread in a roosting situation would be smaller. Such differences in social position may emerge from differences in behavior while feeding and roosting, with certain individuals engaging in more interactions during the day than at night. Such differences among individuals may also emerge from differences in life history (e.g., age, sex, breeding status, etc.) and from differences in movement behavior, a topic that is currently being examined in detail within this system. Investigating the relationship between an individual's spatial and social behavior (Spiegel & Pinter‐Wollman 2022; Webber et al., 2023), may shed light on the mechanisms that underlie individual variation in interactions across different social situations, and consequently on variation in exposure to pathogens (Hughes et al., 2002; Vanderwaal et al., 2016) and information (Cortés‐Avizanda et al., 2014; Spiegel & Crofoot, 2016) and the tradeoffs among them (Romano et al., 2021). Studies of mammals have revealed differences across systems in whether or not the social centrality of individuals is maintained across situations (Gazda et al., 2015; Kulahci et al., 2018; Smith‐Aguilar et al., 2019). Because of the potential implications of the relationship between an individual's social position across situations for population‐level processes, it is important to understand in which systems and when (e.g., different seasons or life history stages) such relationships occur and when they do not.

The contribution of each social situation to the population‐level social structure differed, following our predictions. Co‐flight and diurnal ground interactions are important for determining the number of unique individuals a vulture interacts with and both nocturnal and diurnal ground interactions are important for determining the intensity of interactions. Our finding that vultures seem to repeatedly interact with few individuals while on the ground (e.g., while roosting and feeding), but may have brief interactions with many partners while co‐flying supports our prediction that co‐flight interactions have a lower influence on the strength of interactions than other situations. Indeed, overall, the strength of interactions while flying was lower than in the other two situations (Table S1) suggesting that interactions on the ground are longer and provide more social information for establishing social relationships, compared to co‐flying interactions. It is interesting that both types of interactions on the ground had a similar contribution to the strength of interactions, even though they were measured differently – with diurnal ground interactions measured based on GPS reads every 10 min and nocturnal ground interactions based on a single location each night. Because once vultures arrive at a roost for the night, they do not leave it, a single data point for the night is a good representative of a vulture's location for the entire night, and it allows us to infer which vultures roosted together throughout the entire night. Furthermore, because the strength of interactions was determined using an SRI association index, the number of interactions is scaled within each situation, allowing us to compare across situations.

The strong contribution of co‐flying interactions to the number of individuals one interacts with (degree) relative to nocturnal ground interactions suggests that movement patterns in different situations can influence their impact on social structure. For example, longer ranging movements can result in interactions with more unique individuals relative to short‐distance movements (Spiegel & Pinter‐Wollman 2022; Webber et al., 2023). However, we found that diurnal ground interactions also contributed to the number of unique individuals one encounters, contrary to our predictions. This finding suggests that other mechanisms, in addition to movement patterns, determine who one interacts with. For example, it is possible that carcasses, being an ephemeral resource, attract larger crowds than roosting sites, providing more opportunities to interact with new individuals. This is supported by direct observations of wildlife rangers who estimate that roosts attract 0–20 individuals and up to 70 in extreme cases, and carcasses attract approximately 10–35 vultures, with extreme cases of 80 individuals (personal communications with ranger A. Perez). It is also possible that social preferences differ across situations with a preference for interacting with particular individuals in one situation and different preferences in another situation. Examining social preferences and the relative abundance of feeding and roosting locations and how their distribution in space might influence social interactions can help uncover the mechanisms that underlie the differences we observed in the impact of different social situations on population social structure. While studies of other animals have found that degree (number of unique partners) and strength (intensity of interactions) are not always aligned, examinations of the impact of interactions in different social situations on society structure are limited (but see Dragić et al., 2021; Gazda et al., 2015; Lehmann et al., 2016; Roberts & Roberts, 2016). How each social situation impacts population‐level structure and what are the consequences of the differential impacts of each situation on population dynamics, remain to be examined.

Uncovering how different social situations impact population dynamics can be crucial for conservation and wildlife management actions (Snijders et al., 2017). The population of griffons that we study is of extreme conservation concern (Hatzofe, 2020), therefore, uncovering what types of social interactions are important for structuring the social relationships in the population and identifying which individuals are exposed to different types of information and risks (pathogens and poisoning) can inform wildlife management actions. The prevalence of poisoning as the main mortality reason for griffons and many other vulture species (Ogada et al., 2012) highlights the importance of social foraging and of identifying the social situations that affect social aggregations and individuals' unique roles in these social structures. For example, carcasses can serve as a site for disease spread. Therefore, it would be interesting to examine, if supplementing food at multiple sites simultaneously will spread the vulture population and reduce the number of individuals each one interacts with, potentially slowing the spread of disease and reducing exposure to poisoning. Further investigation is needed to determine the population density that will facilitate vultures utilizing multiple spatially dispersed carcasses simultaneously (Spiegel, Getz, & Nathan, 2013). In contrast, stronger bonds established while on the ground can facilitate the acclimation of introduced vultures and benefit long‐term breeding programs. Future research may help map more precisely when each situation is most important. In addition, more social situations could be considered, such as tandem flights when leaving roosts, which we were not able to identify from the GPS data at the sampling resolution used in our study. Finally, future work can link directly and explicitly the importance of each situation with different fitness consequences.

Uncovering the mechanism by which global population processes emerge from individual interactions can help unravel how societies balance the trade‐off between the costs and benefits of sociality. Considering social interactions within their ecological situation and incorporating the differential impact that each situation has on social structure can uncover previously overlooked causes and consequences of animal social behavior.

## AUTHOR CONTRIBUTIONS

**Nitika Sharma:** Conceptualization (equal); data curation (equal); formal analysis (equal); visualization (equal); writing – original draft (equal). **Nili Anglister:** Data curation (equal); investigation (equal); methodology (equal); writing – review and editing (equal). **Orr Spiegel:** Conceptualization (equal); data curation (equal); formal analysis (equal); funding acquisition (equal); investigation (equal); methodology (equal); project administration (equal); resources (equal); visualization (equal); writing – review and editing (equal). **Noa Pinter‐Wollman:** Conceptualization (equal); formal analysis (equal); funding acquisition (equal); investigation (equal); methodology (equal); project administration (equal); resources (equal); supervision (equal); visualization (equal); writing – original draft (equal).

## FUNDING INFORMATION

Funding for this work was provided by the NSF‐BSF grant: NSF IOS division 2015662/BSF 2019822 to NPW and OS. NA was supported by a stipend from Yad‐Hanadiv.

## Supporting information


Appendix S1.

Data S1.

## Data Availability

Data is provided as part of the supplementary material and the analysis code is available on GitHub (https://github.com/NitikaIISc/VulturesMovementAnalysis_manuscript1).
