# Supplementary material for: Social situations differ in their contribution to population‐level social structure in griffon vultures
Source: Ecol Evol. 2023 Jun 2;13(6):e10139. doi: 10.1002/ece3.10139 (PMC10238758; doi:10.1002/ece3.10139)
Supplement: Supplementary file 1 — Appendix S1. [file ECE3-13-e10139-s002.docx]

**Supplementary materials for: Social situations differ in their contribution to population-level social structure in griffon vultures**

Nitika Sharma^a^, Nili Anglister^b^, Orr Spiegel#^b^, Noa Pinter-Wollman#^a^

^a^ *Department of Ecology and Evolutionary Biology, University of California Los Angeles, U.S.A.*

^b^ *School of Zoology, Faculty of Life Sciences, Tel Aviv University, Tel Aviv, Israel.*

^c^ *Israel Nature and Parks Authority (*INPA*), Israel*

Table S1: Summary statistics for the three centrality measures (degree, strength and PageRank) discussed in the main text, as well as for average strength. Results are shown for each of the three situation-specific networks: co-flight, nocturnal and diurnal ground interactions as well as for the aggregate social network.

| Centrality measure | Social situation | Range  lower | Range  upper | Mean | Median | StdDev |
| --- | --- | --- | --- | --- | --- | --- |
| Degree | CoFlight | 20 | 28 | 26.8 | 28 | 2.0 |
|  | NocturnalGroundInteractions | 8 | 28 | 21. 2 | 21 | 4.1 |
|  | DiurnalGroundInteractions | 13 | 28 | 25.9 | 27 | 3.3 |
|  | Aggregate | 23 | 28 | 27.4 | 28 | 1.3 |
| Strength | CoFlight | 1.1 | 3.5 | 2.1 | 2.0 | 0.7 |
|  | NocturnalGroundInteractions | 1.5 | 9.1 | 4.4 | 4.4 | 2.0 |
|  | DiurnalGroundInteractions | 1.7 | 13.8 | 8.9 | 9.2 | 3.0 |
|  | Aggregate | 5.4 | 23.9 | 15.5 | 16.9 | 4.6 |
| PageRank | CoFlight | 0.020 | 0.053 | 0.034 | 0.034 | 0.009 |
|  | NocturnalGroundInteractions | 0.016 | 0.065 | 0.034 | 0.034 | 0.013 |
|  | DiurnalGroundInteractions | 0.012 | 0.05 | 0.034 | 0.035 | 0.01 |
|  | Aggregate | 0.016 | 0.05 | 0.034 | 0.037 | 0.009 |

**Figure S1: Interactions over time.** Raw number of interactions over time, by month, throughout the study period for each of the three social situations.


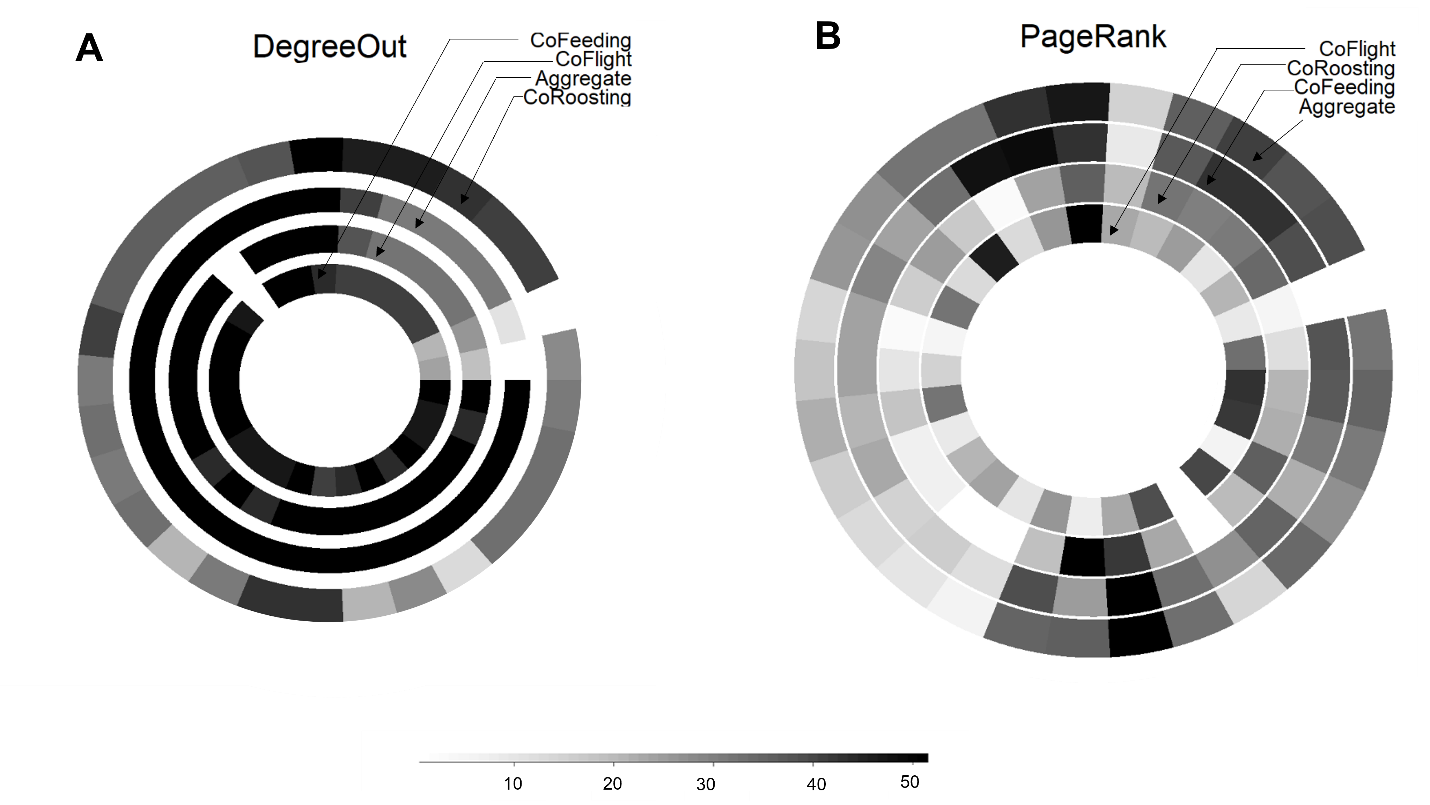


**Figure S2:** **Individuals differ in their social position across social situations.** Annular representation of the Degree (A) and PageRank (B) of 29 tagged vultures in the breeding season of 2021. Each ring corresponds to a social situation. Darker shades indicate a higher rank of degree or PageRank and lighter shades indicate lower ranks. Each slice in the ring corresponds to one individual. Some individuals may be important in one social situation but not in others, see also Figure 4 in main text.
